# Green nanoscience for healthcare: Advancing biomedical innovation through eco-synthesized nanoparticle

**DOI:** 10.1016/j.btre.2025.e00913

**Published:** 2025-08-12

**Authors:** Anjuman Ayub, Atif Khurshid Wani, Suhaib Mohd Malik, Mehvish Ayub, Reena Singh, Chirag Chopra, Tabarak Malik

**Affiliations:** a School of Bioengineering and Biosciences, Lovely Professional University, Jalandhar 144411, India; b Division of Veterinary Biochemistry, Faculty of Veterinary Sciences and Animal Husbandry, Shuhama Alusteng, Sher-e-Kashmir University of Agricultural Sciences and Technology of Kashmir (SKUAST-K), Srinagar, 190006, Jammu and Kashmir, India; c Department of Biosciences, Barkatullah University Bhopal, Madhya Pradesh 462026, India; d Department of Biomedical Sciences, Institute of Health, Jimma University 378, Ethiopia; e Division of Research and Development, Lovely Professional University, Phagwara, Punjab 144411 India

**Keywords:** Nanoparticles, Plants, Cancer, Antibacterial, Wound healing, Sustainability

## Abstract

Green synthesis is an eco-friendly and sustainable approach to nanoparticle production using biological sources such as plant extracts and microorganisms. Unlike traditional chemical methods, it aligns with green chemistry principles by reducing toxic reagents, minimizing waste, and lowering environmental impact. Green-synthesized nanoparticles have shown great potential, especially in biomedicine, for targeted drug delivery, antimicrobial treatments, and imaging applications. Their properties can be finely tuned by controlling size, shape, and composition, supporting applications across electronics, healthcare, and environmental engineering. Characterization techniques are essential for analyzing their structural and functional attributes. However, challenges remain in terms of scalability, reproducibility, and lack of standardized synthesis protocols. The aim of this review is to explore recent advances in green nanoparticle synthesis, evaluate key mechanisms, highlight biomedical and environmental applications, and discuss current limitations. The review also emphasizes future directions and the need for interdisciplinary collaboration to unlock the full potential of green nanotechnology.

## Highlights

• Nanoparticles' size gives them unique physical, chemical, and biological properties.
• Promotes sustainability and reduces the carbon footprint associated with traditional nanoparticle production methods.
• Techniques like UV-Vis spectroscopy, TEM, SEM, and XRD are essential for studying nanoparticle properties and functionalities.

## Graphical abstract

**Figure:**
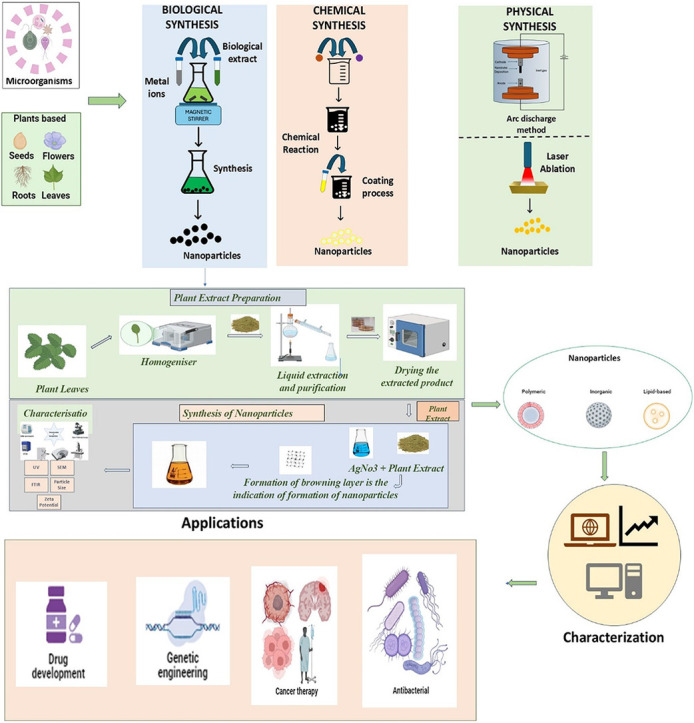

## 1 Introduction

Feynman's 1960 assertion that "there is plenty of room at the bottom" highlighted the vast potential of nanomaterials. His prediction has been realized through advancements in reduction technology, which have enabled innovative methods for synthesizing and characterizing nanomaterials [1]. This progress has led to their extensive application across various sectors. Scientific interest in nanoparticles (NPs) stems from their ability to bridge the gap between bulk materials and atomic or molecular assemblies [2]. Many bulk materials exhibits imposed desirable properties when scaled down to the nanosized particles, with their high aspect ratio, offer enhanced reactivity and efficacy. Researchers have successfully developed nano-sized components for composites and created unique nano-based materials, demonstrating their expertise in this field [3]. Nanoparticles, which are ultra-small particles ranging in size from 1 to 100 nanometres, exhibit unique physical, chemical, and biological properties that are distinctly different from their bulk counterparts. At this nanoscale, these materials demonstrate a higher surface area to volume ratio, quantum mechanical effects, and enhanced reactivity [4]. These properties can be excellently tuned for specific applications through exact control over the nanoparticles' size, shape, and composition [5]. They can be composed of various materials, including metals, semiconductors, polymers, and ceramics. This flexibility enables their use across a broad spectrum of industries, such as electronics, materials science, medicine, and environmental engineering [6]. Nanoparticles have become integral to modern scientific and technological advancements, opening new avenues for innovation in several fields. In medicine, they are utilized for targeted drug delivery, diagnostic imaging, and therapeutic applications [7]. For example, gold nanoparticles are used in cancer treatment due to their ability to absorb light and convert it into heat, selectively destroying cancer cells. In the electronics industry, nanoparticles contribute to the development of smaller, faster, and more efficient devices [8]. Quantum dots, for example, are employed in display technologies to produce more vibrant colours while consuming less energy. In the energy sector, nanotechnology improves the efficiency of renewable energy sources [5]. Nanoparticles enhance the performance of solar cells and batteries, leading to more sustainable energy solutions. Furthermore, nanoparticles are used in environmental applications such as water purification, air filtration, and as catalysts in chemical reactions to reduce pollutants and promote greener processes [9].

Green synthesis refers to the environmentally friendly and sustainable production of nanoparticles. This method utilizes natural resources like plant extracts, microorganisms, and biodegradable materials as reducing and stabilizing agents, avoiding the toxic chemicals and harsh physical conditions typically involved in conventional synthesis [10]. Green synthesis aligns with the broader goals of green chemistry, which aims to minimize the environmental impact of chemical processes by reducing waste, using renewable resources, and enhancing safety and efficiency [11]. Traditional methods for synthesizing nanoparticles often involve hazardous chemicals, high energy inputs, and complex procedures that pose risks to both the environment and human health [3]. For example, chemical reduction methods typically use strong reducing agents like sodium borohydride or hydrazine, which are toxic and can produce harmful byproducts [2]. In contrast, green synthesis methods utilize mild reaction conditions, often at ambient temperature and pressure, and employ natural reducing agents such as plant extracts, which are abundant and non-toxic [12] . This results in more and less harmful byproducts, making waste management easier. The environmental and economic benefits of green synthesis are significant. By using non-toxic materials and processes, green synthesis reduces the risk of chemical exposure and environmental contamination. The use of renewable resources such as plants and microorganisms support sustainable development and reduces dependency on finite resources [9]. Additionally, green synthesis often involves simpler procedures and cheaper raw materials, lowering production costs. The reduction in waste and hazardous byproducts also decreases costs associated with waste treatment and disposal. Moreover, many green synthesis methods operate under mild conditions, consuming less energy compared to traditional high-temperature and high-pressure methods [13].

## 2 Principles of green chemistry in the synthesis of nanoparticles

**Fig. 1:**
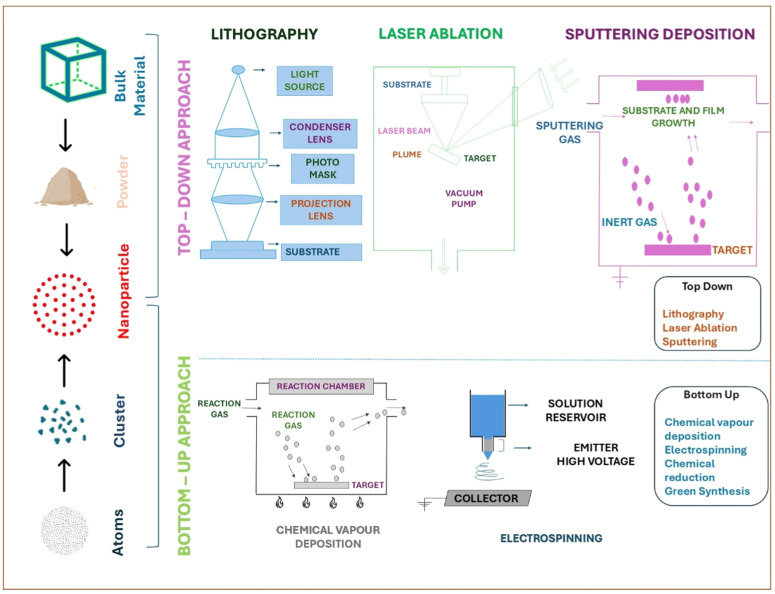
The figure illustrates the two primary strategies for nanoparticle synthesis Top-Down and Bottom-Up. The Top-Down approach begins with bulk materials and breaks them down into nanoparticles using methods such as lithography, laser ablation, and sputtering deposition. In contrast, the Bottom-Up approach assembles nanoparticles from atomic or molecular precursors through techniques like chemical vapor deposition, electrospinning, chemical reduction, and green synthesis. Each method is shown with corresponding instrumentation and processes, highlighting their role in the sustainable development of nanomaterials for biomedical and technological applications.

The field of "Green Chemistry" within the framework of "Sustainable Development" has been extensively explored for under fifteen years. Sustainable development is described as the type of progress that satisfies current needs without risking the capacity of future generations to fulfil their own needs [10]. This concept is particularly crucial for industries related to chemistry due to the challenges of pollution and the excessive use of natural resources [14]. Chemistry has traditionally been viewed as a risky science, with the public often linking the word "chemical" to danger and toxicity. While there are numerous strategies to ease risks through protective gear, failures in these safety measures can significantly increase the likelihood of hazards and exposure [15]. In high-risk scenarios, such failures can result in severe injuries or even. Thus, producing safe and sustainable chemicals and processes necessitates reducing inherent dangers and minimizing the risk of accidents and harm [16]. In the context of nanoparticle synthesis, green chemistry requires three essential elements: an eco-friendly solvent, an efficient reducing agent, and a safe stabilizing material. Various synthetic methods, including physical, chemical, and biosynthetic routes, are employed for nanoparticle production. Chemical methods, although prevalent, tend to be costly and involve hazardous and toxic substances, leading to significant environmental risks [17]. Conversely, the biosynthetic path offers a safe, biocompatible, and environmentally friendly approach, utilizing plants and microorganisms for biomedical applications. This method can use fungi, algae, bacteria, and various plant parts like leaves, fruits, roots, stems, and seeds, which contain phytochemicals that act as stabilizing and reducing agents [18]. Nanoparticle synthesis involves multiple biological and physicochemical pathways, typically classified into bottom-up and top-down approaches, showcasing the diverse techniques available for green synthesis [19]. The bottom-up approach to nanoparticle synthesis involves creating nanoparticles from smaller units such as molecules and atoms, or through the self-assembly of atoms into new nuclei that grow into nanoscopic particles using various chemical and biological methods (Fig. 1) [20] . Conversely, the top-down approach forms nanoparticles by reducing the size of bulk materials into smaller units using techniques like lithography, crushing, splitting, and milling. The stability, shape, and size of nanoparticles can be precisely controlled by adjusting factors such as temperature, pH, concentration of plant extract, metal salt solution, and incubation time [21]. Researchers have reviewed the synthesis of palladium and platinum nanoparticles, detailing the complete synthesis process and their potential applications in diagnostics, biosensors, medicine, catalysis, and pharmaceuticals [10].

## 3 Comparative analysis of green vs. conventional nanoparticle synthesis methods

Nanoparticle synthesis can be broadly categorized into three approaches: chemical, physical, and green/biological methods. Conventional synthesis methods, such as chemical reduction, sol-gel, hydrothermal, and laser ablation, have been widely used for their high yield and reproducibility [22]. However, these methods often involve toxic chemicals, harsh conditions, and complex equipment, which raise concerns regarding safety, environmental impact, and biomedical compatibility [23]. On the other hand, green synthesis offers a sustainable alternative that utilizes plant extracts, microorganisms, and other biological systems as reducing and stabilizing agents under mild, eco-friendly conditions [24]. Although green synthesis minimizes the use of hazardous materials and promotes biocompatibility, it is often limited by batch-to-batch variability, lower yields, and challenges in scale-up and standardization. Reproducibility is one of the key limitations of green synthesis due to variations in the phytochemical composition of biological extracts, which are influenced by seasonal, geographical, and extraction factors. In contrast, chemical synthesis allows for fine control over reaction parameters, enabling consistent results [25]. Similarly, while green-synthesized nanoparticles are typically more biocompatible and suitable for clinical applications, their clinical translation is limited by incomplete mechanistic understanding and regulatory concerns. Conventional nanoparticles, although easier to produce at industrial scale, require extensive purification and surface modification to ensure safety for in vivo applications. The (Table 1) below summarizes the key differences between green and conventional nanoparticle synthesis methods.

**Table 1:** Comparison of green and conventional nanoparticle synthesis methods.

Parameters	Green Synthesis	Chemical /Physical Synthesis
Reducing Agents	Natural compounds (e.g., flavonoids, polyphenols)	Toxic chemicals (e.g., NaBH₄, hydrazine)
Stabilizers/Capping Agents	Plant metabolites, proteins, polysaccharides	Surfactants, polymers, PEG, CTAB
Reaction Conditions	Mild (ambient temperature and pressure)	Often harsh (high temp, pressure, pH)
Reproducibility	Variable due to natural extract composition	High; well-controlled and standardized
Yield	Moderate to low	High
Scalability	Challenging due to biological variability	Easily scalable for industrial applications
Environmental Impact	Minimal, eco-friendly, biodegradable	High, generates hazardous byproducts
Clinical Compatibility	High biocompatibility, lower cytotoxicity	Requires extensive post-processing for safety
Cost	Low, uses renewable and abundant resources	High due to synthetic reagents and energy demands

Despite its eco-friendly and biocompatible advantages, green synthesis of nanoparticles faces significant challenges regarding reproducibility, especially when targeting clinical-grade nanoparticle production [20]. The variability in plant-based extracts, influenced by factors such as season, geography, and extraction method, leads to inconsistent nanoparticle characteristics (e.g., size, shape, surface charge). This heterogeneity presents a barrier to scaling up and achieving Good Manufacturing Practice (GMP) compliance for medical use [17]. For clinical applications, strict control over nanoparticle properties is essential to ensure safety, efficacy, and regulatory approval. To address this, strategies such as the use of standardized phytochemical formulations, pure bioactive compounds instead of crude extracts, and real-time monitoring of synthesis parameters (e.g., pH, temperature, reducing potential) are being explored [16]. Furthermore, machine learning models and automated synthesis platforms may offer future solutions for optimizing and predicting consistent outcomes in green nanoparticle production.

## 4 Green synthesis of nanoparticles (NPs)

**Fig. 2:**
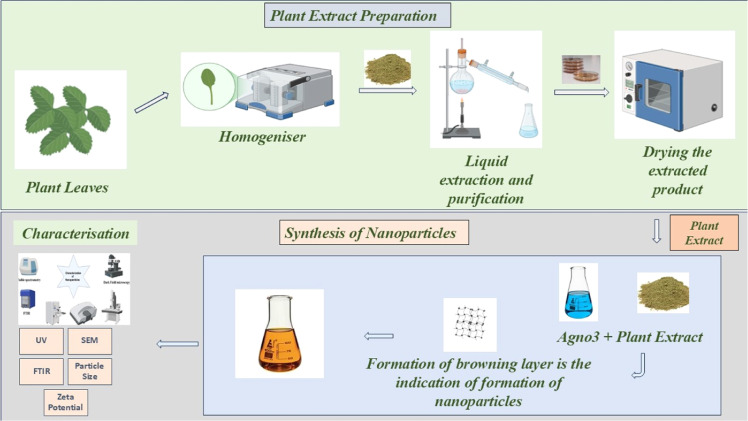
Stepwise schematic representation of green synthesis of nanoparticles using plant extracts. The process begins with plant leaf homogenization, followed by liquid extraction and purification. The dried extract is then combined with silver nitrate (AgNO₃), leading to nanoparticle synthesis indicated by a color change. The synthesized nanoparticles are subsequently characterized using techniques such as UV-Vis spectroscopy, SEM, FTIR, particle size analysis, and zeta potential measurement.

Plant extracts are widely utilized in the green synthesis of nanoparticles due to their rich content of bioactive compounds, such as alkaloids, flavonoids, terpenoids, and phenolic acids. These natural compounds act as both reducing and stabilizing agents [17]. During the synthesis process, plant extracts are mixed with metal salt solutions, where the bioactive compounds reduce the metal ions to form nanoparticles and prevent their agglomeration by capping and stabilizing them [26]. This process generally occurs at room temperature and does not require additional chemicals or high energy inputs, making it both eco-friendly and cost-effective. Numerous plant species have been successfully employed in nanoparticle synthesis (Table 2). For example, the leaf extract of *Azadirachta indica* (neem) has been used to produce silver nanoparticles. Neem (*Azadirachta indica*) leaves are thoroughly washed and boiled in distilled water to extract phytochemicals such as flavonoids, terpenoids, and alkaloids. The resulting extract is filtered to remove solid residues. In the synthesis process, the neem extract is added dropwise to a solution of metal salts, such as silver nitrate (AgNO₃) or gold chloride (HAuCl₄), under continuous stirring at room temperature. A visible color change, such as yellowish-brown in the case of silver nanoparticles, confirms the formation of nanoparticles. To ensure optimal nanoparticle size and stability, parameters like pH, temperature, and reaction time are carefully adjusted during the reaction process, while *Camellia sinensis* (green tea) extracts have been used to produce gold nanoparticles (Fig. 2) [27]. Additionally, extracts from *Magnolia kobus* and *Diospyros kaki* (persimmon) have been used to synthesize platinum and silver nanoparticles, respectively. The diversity of plant species available for nanoparticle synthesis underscores the versatility and potential of plant-based green synthesis methods [28].

**Table 2:** Different sources for green synthesis of nanoparticles.

Source	Name	Type of Nanoparticle	Size (nm)	Biological activity	Reference
**Nanoparticles Extracted from Plants.**					
Plant Source	*Chrysophyllum oliviforme*	Silver	20–50 nm	Antioxidant Anticancer	[29]
	*Acalypha indica*	Silver	20-30 nm	Medical applications	[30]
	*Parthenium leaf*	Gold	50 nm	Scavenges free radicals, cytotoxic against cancer cells, inhibits microbial growth.	[31]
	*Veronica amygdalina*	Silver	2–18 nm	Inhibits gram -positive and gram negative bacteria.	[32]
	*Chenopodium album*	Silver, Gold	10–30 nm	Inhibits fungal growth.	[33]
	*Emblica officinalis*	Gold, Silver	10- 20 nm Ag, 15 – 25 nm Au	Nanofabrication	[34]
	*Swietenia mahogani*	Silver, Gold	50 nm, Au 100 nm	Suppresses bacterial biofilm formation	[35]
	*Terminalia catappa.*	Copper	Cu 370 nm	Reduces microbial infections, catalyzes dye degradation.	[36]
	*Morus alba L*	Copper	20 nm	Effective against *Pseudomonas aeruginosa* and *Klebsiella pneumoniae*, antioxidant activity	[37]
	*Psidium guajava*	Copper oxide	20 nm	Antibacterial activity, reduces blood sugar levels	[38]
	*Zinziber officinale*	Platinum	100-150 nm	Antibacterial against *Staphylococcus aureus* and *Escherichia coli*, free radical scavenging	[39]
	*Morus alba L*	Silver	80-150 nm	Inhibits bacterial pathogens.	[40]
	*Cycas*	Silver	2-6 nm	Reduces microbial infections.	[41]
	*Aloe socotrina*	Zink oxide	15- 50 nm	Anti-microbial	[42]
	*Lippia citriodora*	Silver oxide	15-30 nm	Inhibition of bacterial biofilms	[43]
	*Skimmia laureola*	Iron oxide	56- 350 nm	Enhance root and fruit quality	[44]
	*Jatropha curcas*	Silver	10- 20 nm	Tolerance to drought, environmental stress reduction	[45]
	*O. tenuiflorum*	Silicon dioxide and Titanium dioxide	15- 20 nm	Enhance plant growth and aromatic richness.	[46]
**Nanoparticles extracted from Microbes**					
Microorganisms	*Mycobacterium sp.*	Gold	5-55	Inhibition of tumor growth, apoptosis induction	[47]
	*Pseudomonas sp.*	Silver	410-430	Inhibition of gram-negative bacteria, biofilm disruption	[48]
	*Actinobacter*	Silver	400-500	Antimicrobial effects, bacterial cell wall disruption	[49]
	*Pantoea ananatis*	Silver	320-400	Inhibition of antibiotic-resistant pathogens	[50]
	*Bacillus thuringiensis*	Silver	434.5- 440	Inhibition of bacterial growth, effective against pathogens	[51]
	*Pantoea agglomerans*	Silver Chloride	10–50	Antimicrobial activity against *Staphylococcus aureus, Streptococcus pyogenes, Salmonella, and Bacillus amyloliquefaciens*	[52]
	*Raoultella planticola*	Silver Chloride	10–50	Antimicrobial activity against *Staphylococcus aureus, Streptococcus pyogenes, Salmonella, and Bacillus amyloliquefaciens*	[53]
	*Shewanella oneidensis*	Silver	Varies	Antibacterial activity; potential applications in bioremediation and nanomaterial synthesis	[54]
	*Fusarium oxysporum*	Gold	8–40	Antibacterial and anticancer activities	[55]
	*Fusarium oxysporum*	Cadmium Sulfide	5–20	Potential applications in bioimaging and optoelectronics	[56]
	*Bacillus licheniformis*	Silver	50–100	Antibacterial activity against various pathogens	[57]
	*scherichia coli*	Palladium	2–5	Catalytic activity in dechlorination of polychlorinated biphenyls (PCBs)	[58]
	*Lactobacillus casei*	Zinc Oxide	20–80	Antibacterial activity	[59]
	*Penicillium chrysogenum*	Silver	5–25	Antibacterial activity against *Escherichia coli* and *Staphylococcus aureus*	[60]
	*Cladosporium cladosporioides*	Gold	10–20	Antibacterial and anticancer activities	[61]
	*Saccharomyces cerevisiae*	Selenium	50–200	Antioxidant and anticancer activities	[62]
	*Pseudomonas aeruginosa*	Silver	15–30	Antibacterial activity against various pathogens	[63]
	*Streptomyces sp.*	Gold	5–50	Antibacterial and anticancer activities	[64]
	*Corynebacterium sp.*	Silver	10–20	Antibacterial activity against *E coli* and *Staphylococcus aureus*	[65]

Microorganisms, including bacteria, fungi, and algae, also play a significant role in the green synthesis of nanoparticles. These microorganisms have inherent metabolic pathways capable of reducing metal ions to nanoparticles [66]. They secrete enzymes, proteins, and other metabolites that facilitate the reduction and stabilization of nanoparticles [67]. This biological reduction typically occurs under mild conditions, offering a controlled synthesis environment that can produce nanoparticles with specific sizes and shapes. Several microorganisms have been explored for nanoparticle synthesis (Table 2). For example, the bacterium *Bacillus subtilis* has been used to efficiently synthesize gold nanoparticles [68]. The fungus *Aspergillus niger* is known for producing silver nanoparticles, benefiting from its high secretion of extracellular enzymes. *Algae*, such as *Chlorella vulgaris*, have been employed to synthesize various metal nanoparticles due to their rapid growth and high biomass production [69]. The efficiency of these microorganisms in nanoparticle synthesis is attributed to their ability to produce large quantities of reducing agents naturally. Green synthesis also involves the use of environmentally benign chemicals that are non-toxic and biodegradable [70]. This method leverages chemicals that can effectively reduce metal ions without generating harmful byproducts. These chemicals often include organic acids, amino acids, and natural polymers, which are chosen for their minimal environmental impact [71]. The reactions are typically carried out under mild conditions, reducing the energy required for nanoparticle synthesis, such as organic acids like citric acid and ascorbic acid not only serve as reducing agents but also act as stabilizers, ensuring the nanoparticles maintain their size and shape. Amino acids, such as glutamic acid and cysteine, bring additional versatility through their ability to form stable bonds with nanoparticles, enhancing their stability and functionality. Several green reducing and capping agents are commonly used in the chemical and physical methods of nanoparticle synthesis [26]. For example, ascorbic acid (vitamin C) is an effective reducing agent that can synthesize silver and gold nanoparticles. Other examples include citric acid, which not only reduces metal ions but also stabilizes the nanoparticles by acting as a capping agent [72]. Polysaccharides like starch and cellulose are also used as both reducing and capping agents due to their abundant hydroxyl groups, which facilitate the reduction of metal ions and stabilization of nanoparticles. Capping agents play multifaceted roles in nanoparticle synthesis, beginning with stabilization. They form a protective layer around nanoparticles, preventing aggregation through electrostatic or steric repulsion. Moreover, these agents control nanoparticle size and shape, ensuring uniformity that is critical for specific applications [72]. Capping agents also facilitate functionalization by providing functional groups on the nanoparticle surface, enabling applications in drug delivery, biosensing, and catalysis. Their ability to enhance solubility improves nanoparticle dispersibility in both aqueous and organic solvents. Importantly, green capping agents, especially those derived from natural sources, impart biocompatibility, making nanoparticles safe for biomedical applications [20]. These agents are preferred for their biodegradability, availability, and non-toxic nature, aligning with the principles of green chemistry [73]. The biogenic synthesis of nanoparticles offers a sustainable and eco-friendly alternative to conventional methods. By leveraging the natural resistance mechanisms of organisms, it is possible to produce nanoparticles in a manner that minimizes environmental impact [72]. This approach not only reduces the toxicity of metals but also provides a pathway for the safe collection and utilization of metallic nanoparticles from contaminated environments [74]. The exploration of biogenic methods continues to reveal the potential for developing various types of nanoparticles, each with unique properties and applications, thus contributing to advancements in fields such as medicine, environmental remediation, and materials science [27].

### 4.1 Limitations of using plant extracts in green synthesis

One of the major limitations in green synthesis using plant extracts is the inherent variability in their phytochemical composition, which directly affects the reproducibility and quality of the synthesized nanoparticles [75]. The concentration and profile of active compounds such as flavonoids, terpenoids, polyphenols, and alkaloids can vary significantly depending on several factors, including plant species, geographical origin, seasonal changes, age of the plant, and the part of the plant used (e.g., leaf vs. root) [76]. In addition, differences in extraction methods, solvent polarity, and temperature can further alter the chemical makeup of the extract. This chemical variability leads to inconsistencies in reduction kinetics, capping efficiency, and ultimately the size, shape, and surface properties of the nanoparticles produced [76]. For applications requiring clinical or industrial-grade nanoparticles, such inconsistency presents a significant barrier to scale-up and regulatory approval. To overcome these limitations, researchers are exploring the use of standardized phytochemical formulations, isolated bioactive compounds, and real-time monitoring tools to control synthesis parameters more precisely. Moreover, advances in metabolomics and plant tissue culture are being leveraged to create more uniform bioresource platforms for green nanoparticle synthesis.

### 4.2 Comparative efficacy of green synthesized metal vs. polymeric nanostructures

The antimicrobial efficacy of green-synthesized metal nanoparticles (NPs) is primarily attributed to their physicochemical interactions with microbial cells, such as ROS generation, cell membrane disruption, protein oxidation, and DNA fragmentation [77]. These effects are typically rapid and broad-spectrum, making metal NPs highly effective against both Gram-positive and Gram-negative bacteria [78]. In contrast, polymeric or bio-based nanostructures, including chitosan nanoparticles, alginate nanogels, and PLGA-based systems, often rely on encapsulated antimicrobial agents, intrinsic polymer bioactivity, or pH/temperature-responsive release mechanisms [79]. While these systems generally exhibit lower direct antimicrobial potency compared to metallic NPs, they offer superior biocompatibility, controlled drug delivery, and minimal cytotoxicity, which are advantageous for clinical applications [80]. For example, chitosan NPs exhibit moderate antimicrobial activity due to their cationic nature, which facilitates interaction with negatively charged bacterial membranes [81]. However, their activity is often weaker than that of silver or copper nanoparticles. Overall, metal-based NPs are more effective as standalone antimicrobials, whereas polymeric nanostructures are often preferred for targeted delivery of antibiotics or synergistic therapies.

## 5 Mechanistic insights into phytochemical mediated nanoparticle biosynthesis

The green synthesis of nanoparticles is fundamentally governed by the redox potential and chelating properties of phytochemicals present in plant extracts. These natural compounds, including flavonoids, terpenoids, tannins, alkaloids, saponins, and polyphenols, serve dual roles as both reducing and stabilizing agents during the biosynthesis process [17]. The mechanism involves complex biochemical interactions where functional groups such as hydroxyl (–OH), carboxyl (–COOH), and amine (–NH₂) participate in the reduction of metal ions and subsequent nanoparticle stabilization [82]. For example, flavonoids and polyphenols possess phenolic hydroxyl groups capable of donating electrons to reduce metal ions like Ag⁺ to Ag⁰, initiating nanoparticle nucleation. This redox transformation is facilitated by the relatively low standard reduction potential of silver (Ag⁺/Ag⁰ = +0.7996 V), allowing the reaction to proceed under mild conditions without external chemical reductants [83]. Simultaneously, the oxygen and nitrogen atoms from these biomolecules form coordination complexes with the reduced metal atoms, effectively capping the nanoparticle surfaces and preventing agglomeration through electrostatic and steric hindrance [32]. A well-characterized example is quercetin, a plant-derived flavonoid, which undergoes oxidation of its catechol group to quinone, reducing silver ions while the oxidized quinones strongly bind to the nanoparticle surface, thereby stabilizing them [[30], [83]]. Ascorbic acid, another common plant antioxidant, reduces metal ions via its enediol moiety, while simultaneously acting as a capping agent to control particle growth and shape [84]. The proposed mechanism typically involves four key stages: (i) chelation of metal ions by phytochemicals through their electron-donating groups; (ii) redox conversion of metal ions to their zero-valent state; (iii) nucleation and formation of nanoclusters; and (iv) capping and stabilization by the surrounding phytochemicals [85]. Recent studies have reinforced this mechanism through spectroscopic evidence. For example, FTIR analysis of silver nanoparticles synthesized using *Azadirachta indica* leaf extract revealed the involvement of –OH and –C=O groups in both reduction and capping processes [86]. Similarly, *Camellia sinensis* (green tea) extracts rich in catechins and tannins were shown to reduce and stabilize gold nanoparticles with strong antioxidant potential [87]. These natural compounds not only facilitate the green synthesis but also enhance the functional properties of the nanoparticles, such as antimicrobial, anticancer, and antioxidant activities. Understanding these molecular-level mechanisms is essential for optimizing biosynthesis conditions such as pH, temperature, and extract concentration to tailor nanoparticle size, morphology, and functionality. Thus, phytochemical-mediated biosynthesis offers a sustainable and tunable route for producing biocompatible nanoparticles, bridging green chemistry and advanced nanotechnology [42].

## 6 Characterisation of green synthesized nanoparticles

Spectroscopy techniques are crucial for the characterization of green synthesized nanoparticles. UV-Vis spectroscopy is widely used to determine the optical properties and confirm the formation of nanoparticles by analyzing their absorption spectra. Specific absorption peaks indicate the presence of nanoparticles and provide information about their size and shape [88]. Fourier Transform Infrared (FTIR) spectroscopy helps identify the functional groups on the surface of nanoparticles, revealing information about the biomolecules involved in the reduction and stabilization processes. Raman spectroscopy complements FTIR by providing detailed information about molecular vibrations and chemical composition, essential for understanding the interaction between nanoparticles and capping agents [89,90]. Microscopy techniques provide detailed insights into the size, shape, and morphology of green synthesized nanoparticles. Transmission Electron Microscopy (TEM) offers high-resolution images that reveal the internal structure and size distribution of nanoparticles [91]. Scanning Electron Microscopy (SEM) provides surface morphology and topographical information, making it useful for understanding the texture and aggregation state of nanoparticles [92,93]. Atomic Force Microscopy (AFM) enables the three-dimensional imaging of nanoparticles at the nanoscale, providing information about their surface roughness and mechanical properties. X-ray Diffraction (XRD) is a powerful technique used to determine the crystalline structure and phase purity of green synthesized nanoparticles [94]. By analyzing the diffraction patterns, XRD can identify the crystal structure, lattice parameters, and crystallite size of nanoparticles. This technique is essential for confirming the successful synthesis of nanoparticles with the desired crystalline phases and for detecting any impurities or secondary phases present in the sample [93]. Dynamic Light Scattering (DLS) is employed to measure the hydrodynamic size and size distribution of nanoparticles in colloidal solutions. DLS analyses the scattering of light by particles in suspension to determine their diffusion coefficient, which is then used to calculate particle size. This technique is particularly useful for assessing the stability of nanoparticle dispersions and understanding their aggregation behaviour in different media [95]. The size and morphology of green synthesized nanoparticles are critical properties that influence their functionality and applications. Nanoparticles synthesized using green methods often exhibit controlled size distributions and unique shapes, such as spheres, rods, or cubes, depending on the synthesis conditions and the nature of the reducing agents [96]. Characterization techniques like TEM, SEM, and DLS are essential for accurately determining these parameters, which affect the nanoparticles' reactivity, optical properties, and biological interactions. Surface charge, typically measured as zeta potential, is an important property that affects the stability and dispersion behaviour of nanoparticles in colloidal solutions. A high absolute value of zeta potential indicates good stability, preventing nanoparticles from aggregating due to electrostatic repulsion [97]. Green synthesized nanoparticles often exhibit enhanced stability due to the capping action of biomolecules from plant extracts or microbial sources, which impart a specific surface charge and prevent aggregation (Figure 2).

Green-synthesized nanoparticles exhibit enhanced stability due to the capping action of biomolecules derived from plant extracts or microbial sources. This capping process occurs as the biomolecules, such as proteins, amino acids, polysaccharides, or polyphenols, interact with the surface of the nanoparticles during synthesis. These biomolecules act as stabilizing agents by forming a protective coating around the nanoparticles, preventing them from aggregating or clumping together. This protective coating is crucial because it imparts a specific surface charge to the nanoparticles, which helps in creating electrostatic repulsion between individual nanoparticles. As a result, the particles remain dispersed in solution rather than aggregating into larger clusters [98]. The capping agents also provide steric hindrance, creating a physical barrier that further prevents the nanoparticles from coming together. The combination of electrostatic repulsion and steric hindrance ensures the nanoparticles remain stable in different environments [99]. Additionally, the capping agents often contribute to the functionalization of nanoparticles, making them suitable for various applications such as drug delivery, biosensing, or catalytic processes. The biomolecules also help to improve the biocompatibility and reduce the toxicity of nanoparticles, making them safer for use in biomedical and environmental applications [100]. Functionalization refers to the modification of nanoparticle surfaces to introduce specific chemical groups or biomolecules, enhancing their performance in various applications. Green synthesis methods naturally incorporate functional groups from biological sources, facilitating further functionalization for targeted applications [101]. Characterization of surface modifications is crucial for understanding the interaction of nanoparticles with their environment and optimizing their use in fields such as drug delivery, catalysis, and environmental remediation. Techniques like FTIR, XPS (X-ray Photoelectron Spectroscopy), and Raman spectroscopy provide detailed information on the chemical composition and bonding states on the nanoparticle surfaces, ensuring their suitability for specific applications [102].

## 7 Biomedical applications of green synthesised nanoparticle

Green synthesized nanoparticles offer novel mechanisms for drug delivery, their distinctive surface properties and ability to be functionalized with various drugs. Drugs can be loaded onto these nanoparticles through physical adsorption, covalent attachment, or encapsulation within the nanoparticle matrix [103]. Controlled drug release is achieved through environmental triggers such as pH changes, temperature variations, or specific enzyme interactions [104]. This targeted delivery ensures that therapeutic agents are released precisely at the desired site, reducing side effects and enhancing treatment efficacy. Gold nanoparticles (AuNPs) and silver nanoparticles (AgNPs) are extensively used in drug delivery due to their biocompatibility and ease of functionalization [105] . An example of controlled drug release using environmental triggers is the delivery of anticancer drugs via pH-sensitive gold nanoparticles (AuNPs) [106]. In tumor microenvironments, which are typically more acidic (pH ∼6.5) compared to normal tissues (pH ∼7.4), AuNPs functionalized with pH-sensitive linkers can release the drug specifically at the cancer site. For instance, doxorubicin, an anticancer drug, can be conjugated to AuNPs through a pH-responsive hydrazone bond. Upon exposure to the acidic tumor environment, the bond is cleaved, releasing the drug directly at the target site [107]. This reduces systemic toxicity and enhances therapeutic efficacy. Similarly, silver nanoparticles (AgNPs) have been used to deliver antimicrobial agents selectively in response to enzyme triggers, such as proteases overexpressed in bacterial infections, ensuring precise and potent action [108,109]. Gold nanoparticles can be designed to release drugs in response to external stimuli such as light or heat, making them ideal for targeted therapy. Silver nanoparticles, known for their antimicrobial properties, are also employed in delivering antibiotics and other antimicrobial agents, improving therapeutic outcomes against resistant strains [110].

Several research studies and clinical trials have demonstrated the effectiveness of green synthesized nanoparticles in drug delivery. For example, gold nanoparticles functionalized with anticancer drugs have shown promising results in preclinical studies, enhancing drug accumulation in tumor tissues, and improving patient outcomes [111]. Clinical trials are ongoing to evaluate the safety and efficacy of these nanocarriers in humans, aiming to translate laboratory successes into clinical applications. Green-synthesized nanoparticles have gained significant attention in drug delivery systems due to their eco-friendly production methods and enhanced therapeutic potential. For example, gold nanoparticles (AuNPs) synthesized using plant extracts and functionalized with anticancer drugs, such as paclitaxel or doxorubicin, have demonstrated improved drug accumulation in tumor tissues in preclinical studies. This targeted delivery not only increases the drug's efficacy but also minimizes adverse effects on healthy tissues [112]. Current clinical trials are focused on assessing the safety, biocompatibility, and effectiveness of these nanocarriers in human subjects, with the aim of bridging the gap between experimental success and real-world therapeutic applications. Green synthesized nanoparticles exhibit potent antimicrobial activity through multiple mechanisms. They can disrupt microbial cell membranes, generate reactive oxygen species (ROS), and interfere with DNA replication and protein synthesis [113]. These actions lead to the effective elimination of pathogens, including bacteria, fungi, and viruses, making nanoparticles a powerful tool in combating infections. Studies have shown that nanoparticles, particularly silver nanoparticles, are highly effective against a broad spectrum of microbes, including multi-drug-resistant bacteria [114]. Comparative effectiveness studies highlight their superior performance over conventional antibiotics, especially in dealing with resistant strains. The versatility of green synthesized nanoparticles allows for the development of tailored antimicrobial treatments targeting specific pathogens [111]. For example, a recent study demonstrated that silver nanoparticles (AgNPs) synthesized using *Azadirachta indica* (neem) leaf extract exhibited potent antimicrobial activity against multi-drug-resistant *Pseudomonas aeruginosa* and *Staphylococcus aureus*. The nanoparticles not only disrupted the bacterial cell membrane but also inhibited biofilm formation, showcasing their potential as a powerful alternative to traditional antibiotics in combating resistant infections [115]. Nanoparticles are extensively used in wound healing and medical coatings due to their antimicrobial properties. They can be incorporated into wound dressings to prevent infections and promote faster healing. Additionally, coatings with embedded nanoparticles are applied to medical devices and implants to reduce the risk of microbial colonization and biofilm formation, enhancing the safety and longevity of these devices. Green synthesized nanoparticles serve as excellent contrast agents for magnetic resonance imaging (MRI) and computed tomography (CT) scans. Iron oxide nanoparticles, for example, enhance the contrast of MRI images, allowing for better visualization of tissues and abnormalities [116]. Similarly, gold nanoparticles improve the contrast in CT scans, facilitating accurate diagnosis and monitoring of diseases. Fluorescent and luminescent nanoparticles are employed in bioimaging to visualize and track biological processes at the cellular level [117]. Quantum dots and other luminescent nanoparticles provide bright and stable signals, enabling real-time imaging of cellular functions, molecular interactions, and disease progression. These nanoparticles are essential tools in both basic research and clinical diagnostics [116]. In cancer therapy, green synthesized nanoparticles are used to create targeted drug delivery systems that concentrate therapeutic agents directly at the tumor site. This precision reduces damage to healthy tissues and enhances the efficacy of the treatment. Functionalized nanoparticles can recognize and bind to specific cancer cell markers, ensuring that drugs are delivered precisely where they are needed [118].

### 7.1 Nanoparticles in cancer therapy

Green-synthesized nanoparticles have gained significant attention for their applications in advanced therapeutic modalities, particularly in photothermal therapy (PTT) and photodynamic therapy (PDT) [119]. These therapies leverage the unique physicochemical properties of nanoparticles, such as their ability to interact with light, making them effective in targeting and destroying diseased tissues. The eco-friendly production methods of green-synthesized nanoparticles enhance their biocompatibility, reducing potential toxicity and environmental impact, which is a key advantage over conventionally synthesized nanoparticles [120]. Photothermal therapy (PTT) involves the conversion of light energy, typically in the near-infrared (NIR) range, into heat by nanoparticles. This localized hyperthermia selectively destroys cancer cells or pathogenic tissues without harming surrounding healthy cells [121]. Green-synthesized gold nanoparticles (AuNPs) are particularly suitable for PTT due to their strong light absorption and efficient photothermal conversion [122]. For example, gold nanoparticles synthesized using *Eclipta alba* extract have demonstrated remarkable efficacy in targeting breast cancer cells [123]. These nanoparticles absorb NIR light, converting it into heat that induces cancer cell apoptosis while minimizing damage to adjacent healthy tissues [122]. The biocompatibility imparted by green synthesis ensures that these nanoparticles are well-tolerated by the body, making PTT a safer and more effective treatment option [124]. Similarly, photodynamic therapy (PDT) utilizes nanoparticles to enhance the efficacy of photosensitizers agents that generate reactive oxygen species (ROS) when exposed to light. These ROS cause oxidative damage to cancer cells or pathogens, effectively killing them. Green-synthesized nanoparticles such as silver nanoparticles (AgNPs) or zinc oxide nanoparticles (ZnO-NPs) play a crucial role in PDT by improving the stability, targeting ability, and ROS production efficiency of photosensitizers. For example, zinc oxide nanoparticles synthesized using *Moringa oleifera* leaf extract have been incorporated into PDT systems for skin cancer treatment [125]. These nanoparticles significantly enhance ROS production upon light activation, leading to effective tumor cell eradication with minimal side effects [126]. The integration of green-synthesized nanoparticles in PTT and PDT represents a significant advancement in therapeutic technologies. By combining the inherent benefits of green synthesis, such as reduced toxicity and environmental sustainability, with the precise targeting capabilities of nanoparticle-based therapies, these approaches offer a promising avenue for treating various diseases [127]. As research continues to refine these methods, the clinical potential of green-synthesized nanoparticles in PTT and PDT is expected to expand, paving the way for safer and more efficient treatments [128]. These therapies offer minimally invasive treatment options with reduced side effects. Combining nanoparticles with conventional cancer therapies, such as chemotherapy and radiation, can produce synergistic effects, enhancing overall treatment outcomes [129]. Nanoparticles can increase the sensitivity of cancer cells to these treatments, reduce the required doses, and mitigate adverse effects, leading to more effective and tolerable cancer therapies [130]. In tissue engineering, green synthesized nanoparticles are used to fabricate scaffolds that support cell growth and tissue regeneration. These scaffolds provide a conducive environment for cells to proliferate and differentiate, promoting the formation of functional tissues [131]. Nanoparticles can enhance the mechanical properties and bioactivity of scaffolds, improving their performance in regenerative applications. Nanoparticles can enhance tissue regeneration by providing signals that direct cell behaviour and tissue formation [132]. For example, incorporating nanoparticles into bone regeneration scaffolds can stimulate osteogenesis, accelerating the healing of bone defects. Similarly, in skin tissue engineering, nanoparticles can promote collagen production and angiogenesis, leading to improved wound healing and skin regeneration. These advancements demonstrate the significant potential of green synthesized nanoparticles in advancing regenerative medicine [133].

**Fig. 3:**
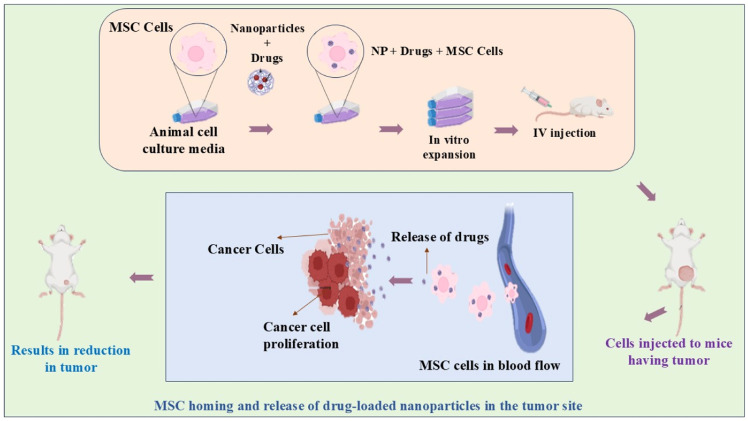
Schematic illustration of drug-loaded nanoparticle delivery using mesenchymal stem cells (MSCs) for targeted tumor therapy. Nanoparticles combined with therapeutic drugs are incorporated into MSCs via culture media. After in vitro expansion, these engineered MSCs are injected into tumor-bearing mice. The MSCs home to the tumor site and release the drug-loaded nanoparticles, leading to inhibition of cancer cell proliferation and significant tumor reduction.

Recent in vivo studies on nanoparticles for cancer therapy demonstrate significant advancements in targeted treatments [134]. For example, lipid-based nanoparticles (LBNPs) [135], such as liposomes and solid lipid nanoparticles, have shown enhanced drug delivery through the Enhanced Permeability and Retention (EPR) effect, which facilitates selective accumulation in tumor tissues while minimizing systemic side effects [136]. An FDA-approved liposomal formulation, Doxil, containing doxorubicin, has been widely used for breast and ovarian cancer, showcasing prolonged plasma retention and reduced cardiotoxicity compared to free drug delivery [137]. In vivo experiments further validate their efficacy, particularly in improving drug bioavailability and reducing adverse effects in mouse models of solid tumors [138]. Another promising area involves prodrug-based nanoparticulate systems that leverage self-assembly mechanisms. These nanoparticles, designed with tumor-specific responsiveness, have demonstrated robust cytotoxicity in in vivo mouse models (Figure 3). For example, dimeric prodrug nanoparticles of docetaxel have shown improved accumulation in tumor sites and better therapeutic outcomes in 4T1 tumor-bearing mice [139]. These systems reduce systemic toxicity while delivering potent anti-tumor effects. In orthotopic patient-derived xenograft models of melanoma, self-assembled nanoparticles co-loaded with chemotherapeutics and photosensitizers have achieved synergistic chemo-photodynamic therapy, demonstrating durable tumor regression and minimal systemic toxicity. Such approaches are paving the way for tailored cancer therapies by combining traditional chemotherapy with advanced nanoparticle technology [136].

### 7.2 Molecular mechanisms of nanoparticle induced antibacterial activity

**Fig. 4:**
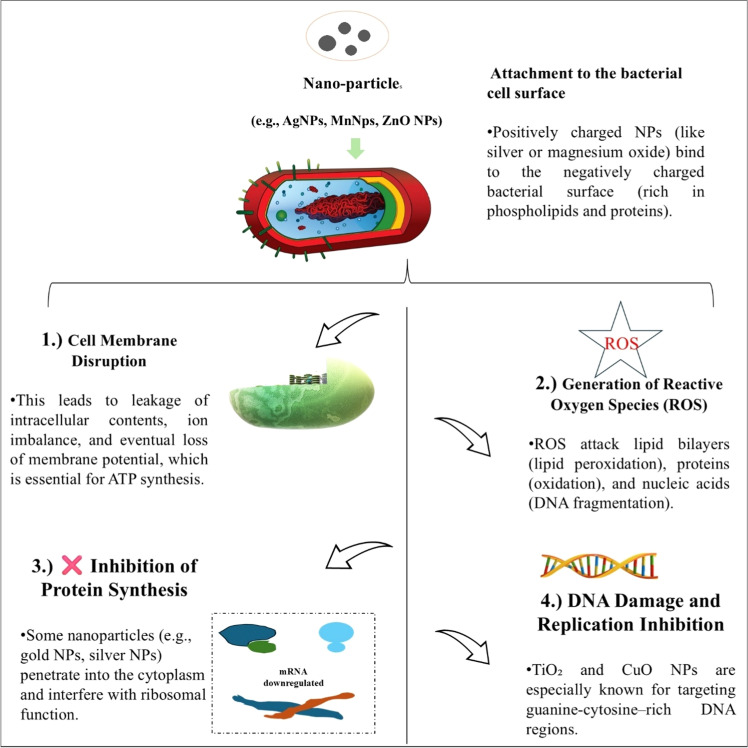
Schematic illustration of the molecular antibacterial mechanisms of nanoparticles. upon interaction with bacterial cells, nanoparticles induce (1) Cell membrane disruption (2) reactive oxygen species (ROS) generation, leading to lipid peroxidation, protein oxidation, and DNA fragmentation; (3) interference with ribosomal function, suppressing protein synthesis; and (4) DNA damage and replication inhibition. These multi-targeted mechanisms contribute to bacterial cell damage and death.

Nanoparticles have emerged as potent antibacterial agents due to their ability to interfere with essential bacterial processes such as protein synthesis, DNA integrity, redox balance, and energy metabolism [140]. Their bactericidal activity is largely attributed to the induction of oxidative stress, metabolic disruption, and interference with regulatory pathways. Metallic NPs like AgNPs, Fe₃O₄ NPs, and CuO NPs are known to trigger the overproduction of reactive oxygen species (ROS) including superoxide anions (O₂⁻), hydrogen peroxide (H₂O₂), and hydroxyl radicals (•OH) which cause oxidative damage to lipids, proteins, and DNA(Figure 4) [141]. This ROS generation activates oxidative stress regulators such as OxyR and SoxRS, which control the expression of detoxifying enzymes like catalase and superoxide dismutase. When the levels of ROS exceed the bacterial antioxidant defense threshold, redox imbalance occurs, ultimately resulting in cell death [142]. In parallel, NPs disrupt bacterial energy production by collapsing the membrane potential through the inhibition of F₀F₁-ATP synthase, thereby halting ATP synthesis and compromising cellular metabolism [143]. Additionally, gold and silver NPs interfere with ribosomal function by preventing tRNA from binding to the 30S and 50S ribosomal subunits, effectively arresting protein synthesis a mechanism that has been confirmed using energy-filtering transmission electron microscopy (EFTEM) and in vitro translation assays [144]. Proteomic investigations using mass spectrometry, two-dimensional electrophoresis, and advanced bioinformatics platforms such as MaxQuant, Proteome Discoverer, KEGG, IPA, and DAVID further reveal significant alterations in bacterial metabolism upon NP exposure [145]. For example, CuO NPs downregulate proteins involved in nitrogen metabolism, electron transport, and substance translocation, while enzymes critical to central metabolic pathways such as GAPDH, isocitrate dehydrogenase, and pyruvate dehydrogenase are notably suppressed, confirming systemic metabolic disruption [146]. Moreover, TiO₂ NPs are known to target guanine-cytosine–rich DNA regions, inducing strand breaks and transcriptional dysregulation, while AgNPs and Au-superparamagnetic iron oxide NPs bind to redox enzymes such as catalase and superoxide dismutase, further depleting the bacterial oxidative stress response and amplifying cytotoxicity [147]. Fig. 4 visually summarizes these multifaceted molecular mechanisms highlighting ROS-mediated stress, ATPase inhibition, ribosomal interference, DNA disruption, and proteomic downregulation offering a comprehensive mechanistic framework for understanding NP-bacteria interactions. Collectively, these findings demonstrate that nanoparticle-induced antibacterial activity is a result of multiple overlapping molecular disruptions rather than a single mode of action. ROS overproduction initiates oxidative stress, triggering regulatory pathways such as OxyR and SoxRS. Simultaneously, ATP depletion due to membrane depolarization compromises energy supply, while ribosomal inhibition halts protein synthesis. Proteomic downregulation further disrupts central metabolic pathways, and direct DNA damage affects gene expression and replication. These mechanisms operate synergistically, leading to widespread cellular dysfunction and bacterial death.

#### 7.2.1 Influence of nanoparticle physicochemical properties on antibacterial activity

Various factors, such as the size, shape, and charge of nanoparticles, play crucial roles in determining their antibacterial efficiency. Smaller nanoparticles, due to their larger surface area, have a greater ability to interact with bacterial membranes. However, some studies indicate that size alone is not the determining factors, as other properties like surface charge and roughness also influence antibacterial activity. The shape of nanoparticles also affects their antibacterial properties [148]. For example, cube-shaped silver nanoparticles exhibit stronger antibacterial effects than spherical or wire-shaped ones due to differences in surface area and facet reactivity. Similarly, prismatic-shaped yttrium oxide nanoparticles have been found to disrupt bacterial cell membranes more effectively than other shapes [149]. Surface roughness is another important factor in nanoparticle-bacterial interactions. Nanoparticles with a rougher surface have been shown to enhance bacterial protein adsorption, reducing bacterial adhesion and ultimately hindering bacterial growth [150]. Zeta potential, or the surface charge of nanoparticles, strongly influences bacterial adhesion and activity. Positively charged nanoparticles, such as Mg(OH)_2_, are more likely to attach to the negatively charged bacterial membrane, enhancing antibacterial effects. Conversely, negatively charged nanoparticles exhibit less bacterial adhesion but still retain some antibacterial activity due to molecular crowding at higher concentrations. To improve the effectiveness of nanoparticles, doping modifications have been explored [151]. For example, zinc oxide nanoparticles doped with fluorine have shown enhanced ROS generation, leading to increased damage to bacterial cells. Similarly, gold-doped zinc oxide nanoparticles exhibit improved photocatalytic activity, increasing their antibacterial efficacy. Titanium dioxide nanoparticles, commonly used in biomedical implants, have been modified to extend their active spectrum to visible light, enhancing their antibacterial properties [152]. These modifications allow for more effective bacterial killing while reducing biofilm formation on implant surfaces. Overall, the antibacterial mechanisms of nanoparticles are influenced by a combination of factors, including size, shape, surface roughness, zeta potential, and doping modifications. These characteristics determine the extent of nanoparticle interactions with bacterial cells, which can lead to membrane disruption, DNA damage, and metabolic inhibition [153].

### 7.3 Nanoparticles in wound healing

Wound healing remains a significant challenge in medical science, making effective wound management crucial. Nanotechnology offers innovative solutions, particularly in regenerative medicine, where self-assembling nanoparticles (NPs) have shown great promise. These nanoparticles, including those made from metals like zinc oxide, gold, and silver, have demonstrated beneficial properties such as reduced in vivo toxicity and potent bactericidal and bacteriostatic activities [154]. With sizes typically in the nanometer range, these NPs enhance wound healing by increasing surface area and improving physiochemical properties. The application of nanomaterials is a rapidly evolving field, contributing to more effective treatments. Among the nanomaterials, metal and metal oxide nanoparticles (NPs) have gained widespread use in biomedicine due to their ease of synthesis, surface functionalization, and enhanced biocompatibility (Table 3) [155]. Gold and silver NPs are among the most studied, with gold being frequently used in drug delivery systems and biosensing applications. Silver nanoparticles, on the other hand, are well known for their antimicrobial and anticancer properties [156]. The antibacterial mechanisms of these metal NPs include altering microbial membrane permeability, generating reactive oxygen species (ROS), and disrupting microbial ATP production and DNA replication, making them highly effective against drug-resistant bacteria [157]. Silver Nanoparticles (AgNPs), for example, have long been recognized for their bactericidal properties and have been used to treat wounds, blisters, and abscesses. Silver nitrate, for example, is still employed to address non-healing chronic wounds. Modern wound dressings incorporating silver nanoparticles are becoming increasingly popular due to their prolonged antibacterial activity without causing complications [158]. AgNPs combined with other materials like collagen or chitosan have been shown to enhance wound healing by preventing bacterial infections. However, the tendency of AgNPs to aggregate can reduce their antibacterial effectiveness, and their toxicity to human cells remains a concern. Similarly, Copper Nanoparticles (CuNPs) have been extensively researched for wound healing due to their excellent antibacterial propertiesn [159]. CuNPs release Cu²⁺ ions, which disrupt bacterial cell walls and membranes, ultimately leading to bacterial death. The antimicrobial activity of CuNPs is size- and concentration-dependent, with smaller particles showing higher efficiency. Moreover, CuNPs generate ROS within bacterial cells, leading to oxidative stress and cell death [160]. Copper nanoparticles also promote angiogenesis by regulating hypoxia-inducible factor (HIF-1a) and vascular endothelial growth factor (VEGF) secretion, further aiding the wound healing process [161]. CuNPs promote wound healing by regulating key signaling pathways involved in angiogenesis. One of the primary mechanisms is the stabilization and activation of hypoxia-inducible factor-1 alpha (HIF-1α), a transcription factor that plays a central role in cellular responses to low oxygen levels. CuNPs prevent the degradation of HIF-1α under hypoxic conditions, enhancing its activity. This activation leads to the upregulation of vascular endothelial growth factor (VEGF), a pivotal molecule in the angiogenic process. VEGF promotes endothelial cell proliferation, migration, and the formation of new capillaries, which are essential for oxygen and nutrient delivery to the wound site [162]. Furthermore, CuNPs directly stimulate the secretion of VEGF from various cell types in the wound microenvironment. The interaction between VEGF and its receptors on endothelial cells activates downstream signaling cascades, such as the PI3K/Akt and MAPK pathways, which drive angiogenesis. This angiogenic response not only facilitates wound closure but also accelerates tissue repair and regeneration [162].On the other hand, Gold Nanoparticles (AuNPs), though not inherently antimicrobial, can be conjugated with other biomolecules to enhance their effectiveness in biomedical applications. AuNPs are biocompatible and can be cross-linked with molecules like collagen, peptides, and growth factors, making them useful in wound healing and tissue regeneration. AuNPs have been shown to improve wound healing by promoting re-epithelialization, granulation tissue formation, and collagen deposition. When combined with polymers or stem cells, AuNPs demonstrate enhanced wound healing capabilities, making them an attractive option for future treatments [163].

**Table 3:** Overview of nanoparticle and Nanoscaffold applications in preclinical wound healing models.

Nanoparticle / Nanoscaffold Type	Preclinical Animal Model	Wound Treatment Procedure	Therapeutic Outcome	References
Silver Nanoparticles (AgNPs)	Murine (Mouse) Model	Full-thickness excisional wound	Accelerated wound healing, reduced bacterial infection, increased collagen deposition, and enhanced re-epithelialization.	[164]
Gold Nanoparticles (AuNPs)	Rat Model.	Skin incision	Improved wound closure, enhanced granulation tissue formation, and promotion of angiogenesis	[165]
Zinc Oxide Nanoparticles (ZnONPs)	Diabetic Rat Model	Excisional wound	Enhanced wound healing in diabetic models, increased cell proliferation, and decreased inflammation.	[166,167]
Chitosan Nanoparticles (ChNPs)	Rabbit Model	Burn wound	Enhanced wound contraction, reduced scar formation, and improved tissue regeneration.	[168,169]
Copper Oxide Nanoparticles (CuONPs)	Murine Model	Full-thickness wound	Increased collagen synthesis, faster wound closure, and antimicrobial effects against various bacterial strains.	[170]
Polycaprolactone (PCL) Nanofiber Scaffold	Rat Model.	Skin graft	Enhanced wound healing, improved cell migration, and vascularization	[171]
Silk Fibroin Nanoscaffolds	Murine (Mouse) Model	Burn wound	Increased wound closure rates, reduced infection risk, and promotion of skin tissue regeneration.	[172]
Graphene Oxide Nanocomposites	Diabetic Rat Model	Excisional wound	Faster wound healing in diabetic conditions, enhanced angiogenesis, and reduced oxidative stress	[173,174]
Hydrogel Nanocomposites with AgNPs	Rat Model	Full-thickness excisional wound	Improved wound contraction, accelerated epithelialization, and potent antibacterial activity.	[175]
Cerium Oxide Nanoparticles (CeONPs)	Murine Model	Burn wound	Reduced oxidative stress, faster wound healing, and improved regeneration of damaged tissue.	[176]

Zinc Oxide Nanoparticles (ZnO NPs) are widely used in cosmetics and therapeutic products due to their biocompatibility and strong antibacterial properties. These NPs disrupt bacterial cell membranes, leading to cell death. Studies have shown that smaller ZnO NPs are more effective against bacteria than larger ones, with their antibacterial activity attributed to their ability to generate ROS [159]. ZnO NPs embedded in biocompatible materials like chitosan hydrogels or collagen dressings have demonstrated both antibacterial and tissue regeneration effects, making them suitable for reducing infection risk during wound healing [177]. In addition to metal-based NPs, peptide nanostructures are an emerging class of nanomaterials in biomedicine, primarily used for drug delivery. These self-assembled structures mimic the extracellular matrix (ECM) and are biocompatible, making them suitable for wound healing and tissue regeneration. Peptide hydrogels, in particular, promote cell attachment and differentiation, helping regenerate tissues such as the liver and bone [178]. The ability to tailor peptide sequences for specific applications further enhances their potential for use in targeted wound healing. Moreover, polymeric nanostructures, both synthetic and natural, are widely used in biomedical applications such as drug delivery and tissue engineering [179,180]. Polymers like polyurethane (PU) and hydrogels have been shown to promote cellular proliferation, angiogenesis, and re-epithelialization, making them useful in wound healing. Gelatin-based scaffolds and fibrin hydrogels are commonly employed due to their biocompatibility and ability to enhance tissue repair. While scaffolds offer mechanical support, hydrogels are more flexible and easier to use due to their injectable nature. Liposomes, consisting of hydrophobic shells and hydrophilic cores, are promising carriers for therapeutic agents in wound healing [160]. They can encapsulate both hydrophobic and hydrophilic drugs, allowing them to penetrate bacterial cells and inhibit biofilm formation. Liposomes can be tailored to enhance drug uptake and absorption by bacterial membranes, making them effective in treating bacterial infections and promoting wound healing [159]. Lastly, lipid nanoparticles (LNPs), including solid lipid nanoparticles (SLNs) and nanosized lipid carriers (NLCs), are gaining attention as efficient carriers for wound healing agents. These nanoparticles protect therapeutic agents from degradation and provide sustained drug release [181]. Their small size allows them to diffuse into biofilms, enhancing their interaction with bacterial cells. SLNs and NLCs have shown advantages over traditional drug delivery methods, making them a promising option for future wound care strategies. Nanotechnology offers a wide array of innovative solutions for wound healing, with metal and metal oxide nanoparticles, peptide nanostructures, polymeric materials, liposomes, and lipid nanoparticles all showing significant potential in this field [12]. These nanomaterials not only promote faster healing but also exhibit strong antimicrobial properties, reducing the risk of infections. Further research is needed to optimize these technologies for broader clinical applications.

## 8 Nanoparticle-mediated genetic engineering

In the last decade, nanomaterials have emerged as an innovative method for delivering biomolecules into plants. Initially, nanoparticle-mediated delivery methods used biolistic delivery systems like gold-functionalized mesoporous silica nanoparticles (MSNs) to transfer DNA and proteins into plant cells [182]. These studies demonstrated the potential of nanoparticles to enter plant cells without requiring mechanical aids such as biolistic, ultrasound, or electroporation. For example, mesoporous silica nanoparticles were able to mediate plasmid DNA delivery in Arabidopsis roots, while layered double hydroxide clay nanosheets enabled the delivery of RNAi molecules to *Nicotiana tabacum* via topical application [183]. Another significant advancement came with DNA origami nanostructures, which facilitated the delivery of small interfering RNA (siRNA) to *Nicotiana benthamiana* [98]. Validation of these methods was achieved through fluorescence microscopy, mRNA, and protein-level quantification, providing concrete evidence of the nanoparticles' success in biomolecule delivery. Carbon nanotubes (CNTs) have also emerged as a powerful tool for delivering plasmid DNA and siRNA into both model and non-model plant species [184]. Demirer and colleagues demonstrated CNT-based delivery of plasmid DNA, resulting in nuclear gene expression, while Kwak and colleagues showed transient YFP gene expression in chloroplasts using fluorescence microscopy [185]. The size, surface charge, and chemistry of CNT particles seem to play a key role in determining their subcellular destinations within plant cells. Positively charged CNTs, functionalized with polyethyleneimine (PEI) polymers, targeted both the nucleus and chloroplasts, while CNTs coated with chitosan polymers appeared to specialize in chloroplast gene delivery [182,186]. These findings underscore the importance of nanoparticle surface chemistry in guiding their movement through various cellular compartments. Notably, both PEI-CNTs and chitosan-CNTs were found to protect DNA cargo from degradation by endonucleases, enabling transient gene expression—an essential feature for future plant biotechnology applications [187]. The ongoing challenge in plant nanobiotechnology lies in understanding which nanoparticle properties, beyond small size, allow for efficient cellular transport. One hypothesis, known as the lipid envelope exchange penetration (LEEP) model, suggests that surface charge may be as critical as size in determining a nanoparticle’s ability to penetrate the cell membrane.

Mesoporous silica nanoparticles, for example, have been observed to pass through the plant cell wall, diffuse through plasmodesmata, and travel via the xylem. It has also been shown that nanoparticle size and surface modifications, such as hydrophobicity, influence their transport within plants [188]. However, the interaction of nanoparticle surface chemistry with complex plant biological structures adds another layer of complexity to designing efficient nanomaterials for plant gene delivery. Nanoparticles must traverse numerous barriers in plants, including the waxy cuticle, dense glycan-based cell walls, and multiple lipid membranes, all while maintaining aqueous solubility and protecting their cargo [189]. Additionally, they must navigate a variety of tissues—leaves, roots, seeds, and callus—to ensure effective biomolecule delivery. These challenges call for cross-disciplinary efforts to optimize nanoparticle design for specific plant physiological and genetic engineering needs. Stable gene expression and transformation are the ultimate goals in leveraging nanoparticles for plant bioengineering. Although CNT-based DNA delivery results in transient gene expression, it offers exciting potential for transgene-free plant engineering [190]. For example, CRISPR plasmids delivered by CNTs could transiently express the gene-editing tool, creating permanent edits in the plant genome without unwanted transgene integration. In addition to nuclear genome targeting, CNTs present an exciting opportunity for plastid engineering. Plant cells contain three genomes, but only the nuclear genome is easily transformable using Agrobacterium-mediated techniques. Since Agrobacterium cannot be used for plastid transformation, biolistic methods are preferred, though they often lead to cell damage and low efficiency [191]. CNT-based delivery offers a more efficient alternative for chloroplast engineering, though stable transplastomic plants remain to be fully realized. Mesoporous silica nanoparticles have already shown success in delivering plasmid DNA into Arabidopsis roots, providing a foundation for extending these methods to plastid transformation. Since the surface chemistry of nanoparticles is tunable, this opens the possibility for delivering a wide variety of biomolecules, including mRNA, single guide RNA (sgRNA), and protein-RNA complexes. Nanoparticles for plant gene delivery are relatively simple to assemble, requiring minimal specialized equipment [191]. For example, polymer-coated CNT constructs can be fabricated in most labs using standard protocols, making them accessible tools for plant biotechnologists. The future of plant nanobiotechnology hinges on the development of systematic guidelines for designing nanoparticles tailored to different plant species and tissues [[187], [191], [192]]. Researchers must investigate how factors like surface charge, particle size, and surface chemistry influence nanoparticle transport and localization within plants. Ultimately, nanoparticle-mediated delivery has the potential to revolutionize plant genetic engineering by offering new ways to overcome the limitations of traditional methods. Stable transgene expression, efficient delivery systems, and the ability to target specific cellular compartments are just some of the many advantages that nanotechnology could bring to plant science in the coming years [[193], [194], [195]].

## 9 Challenges in green synthesised nanoparticle

One of the primary challenges in green synthesis is scalability. While laboratory-scale synthesis is relatively straightforward, scaling the process to industrial levels while maintaining consistency and efficiency proves difficult. Achieving reproducible results with green synthesis methods is challenging due to variations in biological materials, such as plant extracts, leading to inconsistencies in nanoparticle size, shape, and properties [196]. The lack of standardized protocols for green synthesis is a significant hurdle. Different studies often employ varied methods and conditions, complicating the comparison of results and the establishment of best practices. Ensuring the purity of nanoparticles synthesized through green methods is also challenging, as biological materials can introduce impurities that may affect the properties and performance of the nanoparticles [197]. Moreover, the exact mechanisms of nanoparticle formation in green synthesis are not fully understood, hindering the optimization of synthesis conditions and the development of more efficient processes. The introduction of green synthesized nanoparticles, especially in biomedical applications, must address regulatory and safety concerns [198]. Comprehensive studies on toxicity, long-term effects, and environmental impact are required to ensure safe usage. The quality and composition of biological materials, such as plant extracts, can vary depending on factors like geographical location, season, and extraction methods [199]. This variability can impact the consistency and quality of the nanoparticles produced. while green synthesized nanoparticles offer numerous advantages, including environmental friendliness, biocompatibility, and cost-effectiveness, they also face significant challenges [14]. Addressing issues related to scalability, reproducibility, standardization, purity, and regulatory compliance is crucial for the broader adoption and commercialization of green synthesis methods. Continued research and development are essential to overcome these challenges and fully realize the potential of green synthesized nanoparticles [14].

## 10 Future perspective and research directions

Future research in green synthesis is poised to explore novel biological sources and methods to enhance nanoparticle production's efficiency and effectiveness. The discovery and utilization of new plant species, microorganisms, and marine resources could yield a wider array of bioactive compounds, leading to the synthesis of nanoparticles with unique properties [198]. Additionally, advancements in genetic engineering and biotechnology may allow for the modification of existing biological systems to optimize nanoparticle synthesis processes, increasing yield and consistency [197]. Hybrid synthesis approaches that combine green synthesis with traditional chemical and physical methods hold great promise. These approaches could leverage the benefits of green synthesis, such as biocompatibility and eco-friendliness, while overcoming some of its limitations related to scalability and reproducibility [128]. For example, integrating green synthesized nanoparticles with surface modifications through chemical methods could enhance their functionality for specific applications. The field of personalized medicine stands to benefit significantly from advances in green synthesized nanoparticles [[196], [198]]. Tailoring nanoparticles to an individual's genetic and molecular profile could enable highly specific drug delivery systems, improve therapeutic outcomes and reduce side effects. Green synthesized nanoparticles, with their biocompatibility and ability to be functionalized with various therapeutic agents, are ideal candidates for developing personalized treatment strategies [111,132]. Nanotheranostics, which combines diagnostic and therapeutic functions within a single nanoparticle system, represents a cutting-edge application of green synthesized nanoparticles. These multifunctional nanoparticles can simultaneously diagnose, deliver targeted therapy, and monitor treatment efficacy in real-time. Future research will likely focus on optimizing the design and functionality of nanotheranostic agents to enhance their clinical utility, particularly in oncology and other complex diseases [88]. One of the critical areas for future research is the standardization of synthesis protocols. Establishing consistent, reproducible methods for green synthesis is essential for the reliable production of nanoparticles with uniform properties. Developing standardized protocols will facilitate the comparison of results across different studies, accelerate the optimization of synthesis processes, and promote the broader adoption of green synthesis techniques in both research and industry [97]. Comprehensive toxicity studies are crucial to addressing safety concerns associated with green synthesized nanoparticles, particularly for biomedical applications. Future research must systematically investigate the short-term and long-term effects of these nanoparticles on human health and the environment. This includes assessing potential cytotoxicity, genotoxicity, immunogenicity, and biodegradability [113]. Robust toxicity data will inform regulatory frameworks, ensuring the safe and effective use of green synthesized nanoparticles in clinical and industrial settings. The future of green synthesized nanoparticles is promising, with potential innovations in synthesis techniques, advanced applications in personalized medicine and nanotheranostics, and efforts to address current challenges through standardization and comprehensive toxicity studies [132]. Continued research and development in these areas will be crucial for realizing the full potential of green synthesized nanoparticles, paving the way for sustainable and impactful advancements in science and technology [92] .

## 11 Conclusion

Green synthesis of nanoparticles has become critical research focus due to its numerous advantages over traditional methods. This approach employs eco-friendly processes that minimize environmental impact, using abundant and renewable biological materials while avoiding toxic chemicals, thus ensuring safety for both human health and the environment. Aligning with green chemistry principles, this method promotes sustainability and reduces the carbon footprint associated with nanoparticle production. These green synthesized nanoparticles have demonstrated significant potential in a wide array of biomedical applications. They are highly effective in drug delivery systems, improving targeted delivery and controlled release of therapeutic agents. Their potent antimicrobial properties make them invaluable in combating infections and promoting wound healing. Furthermore, their roles as contrast agents in imaging and in cancer treatment especially in photothermal and photodynamic therapies underscore their versatility and clinical relevance. Despite these promising developments, several challenges still hinder the large-scale translation of green-synthesized nanoparticles. A major concern is the lack of reproducibility and standardization, as variability in biological precursors (e.g., plant extracts) can lead to inconsistent nanoparticle characteristics. Factors such as seasonal variation, extraction method, and phytochemical composition affect the size, shape, and surface properties of the nanoparticles, complicating quality control. Another challenge is scalability; while green synthesis is effective at the laboratory scale, translating these methods into industrial processes requires optimization of reactor systems, continuous-flow designs, and supply chain management for bioresources. Furthermore, lower yield and purity compared to chemical synthesis, along with limited long-term stability, present practical barriers to commercialization. The lack of mechanistic understanding of reduction and capping pathways in biological systems also limits the ability to precisely control synthesis outcomes. Additionally, regulatory hurdles and the absence of standardized toxicity assessments impede clinical translation. Comprehensive in vivo studies, lifecycle assessments, and regulatory frameworks tailored to green nanomaterials are urgently needed to ensure safe integration into healthcare and industry. The future of green synthesized nanoparticles remains bright, with ample potential for innovation and development. Advances in green synthesis techniques, including the discovery of novel biological resources, bioreactor-assisted synthesis, and hybrid green-chemical strategies, are expected to yield nanoparticles with improved stability, targeting efficiency, and functional versatility. These innovations will expand the scope of green nanomaterials in personalized medicine, regenerative therapies, and nanotheranostics, paving the way for more effective and tailored healthcare solutions. To fully realize this potential, interdisciplinary collaboration is essential. Integrating expertise from chemistry, biology, materials science, and medicine will accelerate the development of more efficient synthesis protocols, deeper mechanistic insights, and broader biomedical applications. Overcoming current limitations through standardization, real-time monitoring technologies, and sustainable upscaling will ensure that green nanotechnology can transition from promising laboratory research to transformative real-world solutions. Continued research, innovation, and cross-sector collaboration are therefore crucial to unlocking the full potential of green synthesized nanoparticles, ultimately driving meaningful advancements in science, technology, and global healthcare.

## CRediT authorship contribution statement

**Anjuman Ayub:** Writing – review & editing, Writing – original draft, Visualization, Validation, Software, Resources, Methodology, Formal analysis, Data curation, Conceptualization. **Atif Khurshid Wani:** Writing – review & editing, Visualization, Validation, Methodology, Formal analysis, Conceptualization. **Suhaib Mohd Malik:** Writing – review & editing, Visualization, Validation, Methodology, Formal analysis, Conceptualization. **Mehvish Ayub:** Writing – review & editing, Visualization, Validation, Methodology, Formal analysis, Conceptualization. **Reena Singh:** Writing – review & editing, Visualization, Validation, Resources, Methodology, Formal analysis, Data curation, Conceptualization. **Chirag Chopra:** Writing – review & editing, Visualization, Validation, Supervision, Methodology, Investigation, Formal analysis, Data curation, Conceptualization. **Tabarak Malik:** Writing – review & editing, Visualization, Validation, Supervision, Methodology, Investigation, Data curation, Conceptualization.

## Declaration of competing interest

The authors declare that they have no known competing financial interests or personal relationships that could have appeared to influence the work reported in this paper.

## Data Availability

The data that has been used is confidential.
